# Address before the Graduating Class of the Medical Department of the University of Buffalo, on the Occasion of the Annual Commencement, February 22, 1870

**Published:** 1870-04

**Authors:** Walter Clarke


BUFFALO

### Medical and Surgical Journal.

VOL. IX. APRIL, 1870. No. 9.

### Original Communications.

ART. I.—*Address before the Graduating Class of the Medical Department of the University of Buffalo, on the occasion of the Annual Commencement, February 22, 1870.*

By WALTER CLARKE, D. D.

GENTLEMEN OF THE GRADUATING CLASS:

For some reason, that to me is as mysterious as medicine, your instructors have changed the custom of former times, and, instead of selecting one of their own number to address you this evening, have taken counsel of ancient example, and asked me to give them of my oil. Whether it was some discovered defect in their lamps, which I do not, you will observe, insinuate; or whether, having experimented on former classes with wisdom dispensed in allopathic measures, they conclude to change the treatment, and administer it to you to-night in pellets; or whether they had seen that in your conduct which suggested the need of a theological tonic—unaccustomed as I am to the dissection of a doctor's reason, I leave these otherwise interesting inquiries among the unsolved problems of the day. And yet, to justify the sagacity that, in its straits, sought access to my can, I ought to tell you that, if the customary wicks did decline to burn on this occasion, and your teachers wanted the aid of one whose large experience of like disaster made him sympathetic in the extreme, they rang at the right door, when, of a cold night in December, they called at the parsonage in Washington street.

But I must give you gentlemen, an address of congratulation, and, if you will receive it from a well disposed stranger, of counsel to-night. What shall it be? Of what shall I speak to you on the joyful occasion of your leaving the class-room for the patient, and exchanging the lecture for the fee? You have had your fill, I presume, of therapeutics and materia medica; and, were it otherwise, I could not help you. I do, indeed, deal much with skeletons; but as they are of a kind that handle the living, and do not allow the living to handle them, I dare not venture, for your sakes, to expose you to them, or for their sakes, to expose them to you.

Would it assist you to enter more hopefully upon your profession, if you should, for a few moments, look at it through other than doctors eyes? Perhaps it was to give opportunity for friendly suggestion from without, that one not of your guild was chosen to deliver this parting address.

To us, your seniors, who have served in our several callings till we understand their demands, either of the three great professions, and yours especially, is a very different thing now from what we imagined it when, with youthful fancies, we compared their merits and made election of our favorite. Then we were like girls yielding to the solicitation of their suitors. What we accepted as servants, we have as masters. History has seen tyrants innumerable, but none that were more exacting than a profession, whether it be medicine, or divinity, or law. And it is well, young gentlemen, that, if you have had till now romantic conceptions concerning the life upon which you are so soon to enter, you should lay them off at the threshold of the coming reality. However they may help the student, they only encumber the practitioner. A profession, especially in our country and age—and your profession more than any other—is not an easy chair, in which you can lounge for life; nor a cushioned conveyance, in which you can roll on smooth pavements to wealth and greatness; nor a limber lackey, whom you can master and employ at your will. From the time you enter it, you are its slaves. It will feed or starve you; give you work or deny it; set you down where it will; help or hinder, comfort or torment you; lean over your chair by day, your pillow by night; listen to no complaints; accept no denials. Sleep, sickness, fatigue, the duties of society; the calls of literature; the cares of home; considerations that avail with other tyrants, will go for nothing with yours. I am glad, gentlemen, that there are better things to be said of your profession than that it is supremely despotic. And if I remind you of its exactions, it is not to depress your enthusiasm, but only to prepare you for a more courageous, and therefore more hopeful beginning. You cannot negotiate with your calling. If you take it at all, you must take it as it is. It has no need of masters. What it asks is servants. You have given attention to its lessons; equipped yourselves with its qualifications; finished your probation, and are ready to enter on your work. No, gentlemen, not yet. One thing more, or you will incur early disappointment and ultimate failure. Can you devote yourselves to your profession? As if it were a waiting altar, can you lay upon it the consecrated body, the consenting mind? Not to faith alone nor to virtue alone, nor to the service of Christ alone, is sacrifice the only gate of entrance. We must accept our various callings upon the same inexorable conditions, if we would enter them with hope.

It should be a matter of serious consideration with every young man, crossing the threshold of an untried profession, that, whatever he is to be in after life, he is to become by means of his chosen calling. The several pursuits in which men engage are so many stairways, on which, if they slope upward, we are to climb to greatness; if they bend downward, grope to darkness and contempt. Each of us may select on which to set foot; but the calling once chosen, our fate is in it, and we must be what our work shall make us. You have selected for yourselves, gentlemen, one of the noblest of all human arts; and wherever it may lead you, it has every needed appliance with which to energize, develop and ennoble you. Summoning to its service all the hidden powers of intelligence and sound judgment, calling into daily exercise the susceptibilities of a warm and generous heart; offering abundant scope for personal improvement and a boundless field for professional research; enlisting in its tasks the senses of sound, and sight, and touch, unfolding in this way the whole man and employing those united powers on the best subjects and for the best ends; if there be a calling in the whole round of human activity which can make its subjects well developed and great, and ought to do it, it is the practice of medicine. Behold, then, your opportunities, young gentlemen, and with them your limitations also. If your powers are to expand at all, they must expand like growing vines on the trellis to which they have been made to cling. Let the sculptors be great with the chisel, the painters great with the brush, the astronomers great with their glasses, the statesmen great in council, the preachers in discourse, the warriors in arms, the financiers in incomes; for you it is reserved to be great only as doctors. And you may well be content with your lot in this respect, though it offer you but a single pathway to distinction. It has noble work to be achieved, noble attainments to be reached, and noble satisfactions with which to reward its pupils. But beneficent as is your calling, and rich in rewards for the worthy, it is at the same time a steep ladder, at whose feet lie all the idlers—whose summit is accessible only to those who have the energy and the will to climb.

There is a subtle and a very powerful temptation, gentlemen, which, in common with all who go into professional life, you will soon encounter, and which, unless you detect and dispel it in time, will possess and paralyze you. I mean the disposition to regard your calling as an unpleasant necessity—an irksome but indispensable way of earning your bread. Nothing in life is more imperious, perhaps, than this business of earning one's bread; at the same time, nothing but vice more effectually belittles us, than seeking bread, as the supreme and all-comprehensive aim. The characteristic of great minds is, that, however unpretending their work, they idealize, and in that way, ennoble it. They are not makers of anything—they are fathers. The products of their heads and hands, if not their children, are their deities or their brides. Here is the distinction between what is true art and what is only a trade. Here is the dividing line between genius and machinery. The man who sketches landscapes, carves statues, constructs poems, links together the notes of music, having in mind, not the beauty and joy of his work, but only the market and the prices, has lost the last spark of his manhood, and become an engine for coining copper. He, on the other hand, who hammers the hot iron, turns the heavy clod, sharpens the axe, or swings the scythe, attracted not by the coming compensation, but by the consciousness of creative power—he is the true artist. There is little in your profession, gentlemen, your tedious vigils, your toilsome rides, your endless and wearisome routine, to keep alive your enthusiasm. And if you fix attention mainly on your fees—these are not the fuel with which ambition burns. But if you wed medicine as an art, love it for its own sake, seek your chief reward in finding its secrets and developing its powers; if it be to you, like virtue, its own sufficient reward—like beauty, its own supreme attraction—you will lose the drudgery, gain the bread, and, what is far more, experience the joy of life-long fellowship with an ennobling art.

You are greatly superior to those who have gone before you, gentlemen, if you do not need to be reminded, in this farewell interview, of the unwisdom of regarding the philosophy of your profession as already complete and immutable. The oldest of all the sciences, yours is at the same time the least mature. Not because it is a duller scholar, but because it has a longer lesson. Inside the frame of any living babe there are ampler treasures of knowledge, whether we have regard to mass, variety or practical worth, than the telescope can find within the whole circuit of the skies. Long ago the astronomers had read all that nature had inscribed for our information on the shield of Hercules, the disc of Neptune, or the forehead of the Northern Bear; but when will physiology find the bottom of any cell, exhaust the contents of any drop of blood, or extract its secrets from an atom of the brain? The mysterious element of life that operates unseen in all the phenomena which you are called to inspect, is enough of itself to postpone to another century the completion of an accurate code of disease and cure. In the march of modern science the van is given to mathematics—the rear to medicine. Nor is the humilition of an incompleted science your only inconvenience in this behalf. Ours is an age of dominant dogmatism; and dogmatism is as popular as it is rife. Go where you will to find employment, you will encounter a score of confident boasters for one who is desirous still to inquire. We are accustomed to congratulate our age as the happy era in history when, for the first time, the oracles of superstition have abandoned themselves to silence and forgetfulness. But they are mute only to give place to the noisier clamor of the sybils of science. For both these reasons it will be difficult for you, young gentlemen, to stand beside mathematicians, mineralogists and experts of all kinds, and, while they assume the style of assurance, exhibit on your part the modesty of a profession which yet has something to learn. "This is nitre," says the chemist; "give it three parts of sulphate of iron and five of common salt, and, without any possibility of another issue, it will become nitro-muriatic acid." And "this is the ninth digit," says the mathematician; "add to it five and two and twenty, and you will have for your result, and no mistake, a score, half a score, and six." Alas, for you! You are a doctor, and it is your turn next. Can you tell what to mix with this typhoid fever, and in what proportions to make of the compound soundness and strength?

It will only aggravate your disadvantage if you condescend to take example of ancient models, and conceal imperfect knowledge under the mask of wise looks, impressive silence, or unintelligible phrases. There is a time in early morning when from scarcity of light, the effigies of the cornfield seem so many veritable giants. So in the dawn of popular intelligence, the face of an owl may be supposed to possess the ken of an eagle. But you were born, gentlemen, too late in the nineteenth century, to hope for shelter or assistance from professional airs. What, then, shall you do? I give you my advice. Let those who imagine that they have attained to premature omniscience recite their formulas and do all the dogmatizing. For yourselves, be content to know what you can know, and for the rest substitute for easy pretension, patient and protracted research. Nobody expects you to know everything about disease or its cure. Nobody asks you to outrun the revelations of your slow paced art. So long as your patients shall discover that you are students and not pretenders, explorers and not oracles, builders of knowledge, and not boasters merely, they will confide in you, honor you, and pay you. Nor is the immaturity of the medical art a ground of humilation alone. In another view it is an argument of joy. So long as there is ore in the cave the miners must pursue their work. And if there be weariness in the labor, there is, also, be it remembered, wealth in the result. That will be a sad day to any of the sisterhood of study—to astronomy, to chemistry, to art in either form—when the goal is reached, the material gathered, and there is nothing left but to remember and rehearse and lounge eternally in the lap of the unproductive past. "If the Almighty should offer to me," said Lessing, "in his right hand truth and in his left hand the search for truth, and command me to make election between them, I would throw myself eagerly to the left and say: Father, grant me this and keep the other for Thyself." Of all the fields of scientific research open to the student of to-day, I know of none that offers more attractive, more important, or more certain rewards, than that to which you are devoting your lives. Every new patient is a new study. Every recurring symptom, a new revelation. To explore to its bounds, the action of temperament upon disease and its antidotes; to trace the influence of imagination as it throws auroral light upon the inner life, tinging with prismatic hues the symptoms alike of sickness and health; to follow to their source the ten thousand hereditary predispositions and causes; to determine the sanitary force of the passions and the will; to estimate with accuracy the influence of habit and outward condition; to detect the union of similar, the transposition of different types, and track the myriad changes, through which disease may pass: to push inquiry thus on a thousand inviting lines, and come back to your Alma Mater, from year to year, as the spies of ancient Israel, bearing clusters from the land of promise. There is nothing in being able to say four times four is sixteen, or so far off is Venus, or so much hydrogen with so much of something else is water, to compare with this. Be it so, that like a faithful guardian, your profession gives you only an allowance, so that compared with heirs, and especially with prodigals you can be neither lavish nor proud. Your estate is ample; your title perfect, and when the time comes, medicine shall be pronounced not the oldest only, but the richest of all the legatees of science.

The teachers of your profession have kindly reminded us whose business is the cure of souls, and I confess there was need of this warning, that in our too exclusive attention to the spiritual part of man, we overlook the fact that he has a body as well. Into all the mental moods and aspects, they say, somewhat of the flesh will filter, and the flow of religious feeling, like the current of river or brook, will catch the tinge of the earth through which it is compelled to pass. May I not suggest, young gentlemen, a kindred peril on your side. The tendency of all your studies, as you have doubtless perceived, is to account for whatever appears in body or brain by attributing it to a vital force. In other words, if theology is tempted to ignore the physical part of man, in its too engrossing attention to the spiritual, physiology and anatomy are in danger of overlooking the soul in their intent contemplations of its vesture. It cannot be questioned that the body contributes largely to what is contained and experienced in the mind. Is it not equally true, that the mind contributes much of what it suffers and enjoys to its neighbor the body? And if they whose profession devolves on them the cure of what is spiritual in man, err when they refuse to take into account the influence of his senses, sensations, and nervous tissues, do they err less, who, having in charge his body, treat it as if it were complete in itself. I know not which the future would have cause more to lament, were we of the clergy to forget that men have bodies, or you of the medical class, to ignore the presence of their souls. We must do neither; but standing on opposite sides of the same object, we must penetrate it—you, till through the material you discern the spiritual—we, till through the spiritual we behold the material, for so only can we reach and heal the whole man.

I shall possibly do you a service to-night, by drawing your attention, for a moment, to a fact familiar to men outside of your profession, familiar perhaps to yourselves, that the powers which a physician can ill afford to lose, are those which practice is most likely to blunt—I mean his large and quick sympathies. By a well known law of nature, familiarity with suffering gradually abates the sense of it. By another law, excessive sympathy destroys the power to help the distressed. By the operation, I doubt not in many cases by the unperceived operation of these two causes, many a physician loses in practice as fast as he gains in skill. Occupied with symptoms, indifferent to sufferings, intent, persistent and resolute, before he knows it, skill and purpose are begirt with an investiture of ice, and the practitioner handles with the same indifference his living patient and his lifeless tools.

Be assured, gentlemen, you will miss many of the most precious possibilities of your profession—if of oversight, or consent or any other cause, you cease to be medical men, and become only medical figures in stone. For yourselves, sympathy will be ever a new sense with which to explore disease; while for your patients it will be ever a new medicine with which to mitigate and heal it. What

GOETHE said of the sense of beauty, has equal application to the point in hand. "Man," wrote that greatest of the German poets, "is so inclined to deliver himself up to the commonest things—mind and sense are so easily blunted to the impression of genuine beauty, that we ought in every way to preserve the power of feeling. And since no one can utterly lose the faculty, neglecting to use it is the only reason that so many find pleasure in ugliness. We ought, therefore, at least once a day, to listen to a little song, read a good poem, view a fine landscape, and if it were possible to do it, speak a few sensible words." Not for a day will one of you allow his surgical instruments to be rusted or dull. It will pay you, gentlemen, to keep your sensibilities in equal repair.

Have I need, on an occasion like this, to say a word regarding that grossest and most brutish of all the forms of professional apathy—bluntness and ill-breeding. Some men are born boors. They not only neglect good manners—they even despise them. We have a few such bumpkins (I speak in a catholic and not a sectarian sense) in our pulpits, sorrowful to say. It is reported that some of them have strayed into your profession, and got access to the chambers of the sick. To how many a timid child, or delicate woman, therefore, is the chief terror of disease, the anticipated tread of the heavy foot, sight of the slovenly dress, touch of the wooden fingers of the awkward but inevitable doctor. No physician is under moral obligation, perhaps, to be an Apollo. Nature having originally two gifts, beauty, and the admiration of beauty, consulted our wishes, and lavishing the former on the softer sex, left us to get on as we could with the latter. It may be impossible for some of you, gentlemen, I say it with becoming commiseration, to be handsome. If so, and birth denied you the boon of beauty, do not, I pray you, by making yourself fops, demonstrate that it withheld brains also. I said that we of the rougher sex were early disinherited of beauty. No, I correct the false suggestion. Denied the grace of person, we were compensated by the higher charm of possible gentleness of mien. Accordingly there is no deformity of figure, and no defect of education, and no blemish that time can inflict on our sex, that may not be covered and made beautiful by the enamel of elegant manners. We need well-bred men everywhere—in Congress, in the Court room, in the street cars. We need them especially in the sick room. You have adopted of late, the admirable device of sugar coating. Your patients like it. Many a disgusting drug, the dread of our brave ancestors, is palatable now by reason of its smooth and saccharine surrounding. Would it do any harm to extend the charming experiment, and try its powers on the acrid practitioner, as well as on the offensive and distasteful pill?

It was understood, I believe, between the parties negotiating for this address, that whatever liberties of discourse I might for the moment arrogate, if I went the length of heresy even, my body should still be safe from the hands of the dissector. I venture, therefore, in a timid way indeed, to offer you a hint or two on the subject which, as an observer, I perceive is becoming to your profession more and more difficult to adjust. Do not be agitated with premature alarm, gentlemen. I am not about to speak of a falling scale of fees, or the old habit of experimenting on clergymen gratis. Of this latter custom I will only say in passing, *esto perpetua.* I refer to your relations as of the orthodox school, in the regular succession, —the ancient practice and the true church, towards dissenters of all classes, homœopaths, hydropaths, electricians, and what nots. We theologians have had for the last nineteen centuries rather a lively time with this sort of people, who prefer to the homely old ark of safety the jaunty little cockboat of doubt. And we have tried two different methods of intercourse, and reached, at length, what we venture to commend to you, as the ultimate philosophy on this subject. At first we took the ground that tares were not wheat, nor half as good as wheat, and that our office as growers of the latter grain included a function of grubbing at the former. Quite a number of Christian centuries have made record of our diligence in uprooting the tares of heresy, and casting them into the fire to be burned. A venerable gentleman in a distant city, waiting the decree of infallibility from one quarter, and of mortality from another, and more and more fearful every day that the messenger from the swift Styx will get the start of the envoy from the slow Council, continues this method, and occupies his leisure, we are told, in penning Latin paragraphs, to be used as mattocks in digging out of the soil of Christendom several nations, of no ignoble attainments, and of very respectable size.

But study, disappointment, and waste have revealed to us in these latter days the somewhat important fact, that what we call heresy is apt to be made up of two very different elements. And though in the admixture, truth may be to falsehood as two grains of wheat to two bushels of chaff, there are chickens in every land, that will judge of the pile not by the refuse which they cast under their feet, but the barley corn which they carry home in their crops.

The devotees of science, whether secular or sacred, go through their fields like rapid reapers, eager to gather at every advance, new armfuls of the ripened grain. What wonder if, in their haste, they drop an occasional stalk, or leave behind at evening, a neglected and unmissed sheaf. Nay, what wonder if next day, the gleaner discovers these mistakes, and notifies the world that he has biscuits to sell, as good as any to be found in the market.

The oystermen are a useful race, but in their exclusive attention to the pulpy, they sometimes overlook the wealth that lies in the solid part. When we desire a classic dish, the rich fry, the steaming stew, the economic and deceitful scallop, we go hopefully to these lords of the count and the can, little dreaming that perhaps they have left among the shells that make the wealth of the scavenger, pearls worth tons of their precious and popular mollusks.

Thus wherever he wields sickle and wain, the reaper Science is father of the gleaner Dissent. Nature, lavish in one respect, is frugal in another. Pillage as we will her bivalves, we may not pass by her pearls. Whatever the orthodox leave, the heretics will pick up. Pondering these facts the wisest theology of our day has discovered that its relations to the thousand schools of dissent are only partially hostile, and that in another and higher sense, they are complementary and fraternal. Whatever in books, and schools, and systems, is chaff, we surrender to the flames or give freely to the wind. But whatever is wheat, if there be but two berries of it, we take as of our inheritance, and put it in our pile. To be orthodox is to be heir to all truth, and ready to lay hold of it wherever it is found. We have, therefore, changed the tactics of our fathers, and whereas they denounced much and investigated little, we anathematize rarely, and inquire the more. Would it offend you, gentlemen, if without questioning your title to pre-eminence, I should hint this evening that your best way to disperse all other styles of practice, and deserve and win the whole field to yourselves, is b search and comprehension, and not by censure and repulsion. A man comes to you for treatment; his disease is complicated, the effect of great abstinence on the one hand, and great excess on the other. Call his body a transport for carrying provisions, and it has been overloaded in every voyage for the past fifteen years. Consider it as a garment, and in all that time it has never entered the tub. You lift the hatches, pour into the hold a preparation of drugs and commend him to a time of medicine, or the medicine of time. Having tried your course sufficiently, he incloses a five dollar note to you, inquires for a water-cure, goes to it, aud in two months comes home minus his distemper and a large roll of greenbacks. What cured him? An occasional wash, and the diet of five men, diminished by four. Would it not be as wise, and in the long run, as profitable a use as you could make of this unhappy case, if, smothering in your own bosom your dislike of hydropaths, you should resolve never to furnish them with another subject, and when the next man, with similar symptoms, rings at your office door, give him this prescription:

*Submerge quotidie in aqua callida et plena*;

*Attrite vehementer cum manu et cum crash towels*;

*Pasce frugaliter, cum bove sano, cum pane cibario, et cum tuberibus tostis*;

*Reste ad domum, et in fine, pay to your orthodox doctor what it would cost to get cured at a water cure.*

Eclecticism and comprehension are the indispensable elements of orthodoxy, whether in physic or metaphysic, in the care of the body or the cure of the soul. On the other hand, the components and characteristics of dissent, wherever you find it, are bigotry and seclusion.

When the homœopath says *similia curantur*, if he would stop there, his motto would be as comprehensive as the science of cure. But when he slips into his rut and adds *curantur similibus*, he imitates the French tanner, who to his majesty, declaring that the city must be begirt with armor-defying walls, answered: "Yes, sire, and of my good leather."

The woodman who delivers hickory only by the cord, must not complain of the grocer because he sells it in kindlings at a penny a bunch. Let him cease denouncing, set up in his yard a circular saw, and consent to furnish fuel in both forms, and the grocer who would never succumb to the scold, will quickly give place to the rival. The forms of practice, different from yours, which are any thing better than quackeries and shams, will continue to thrive till opposed with something more than the votes of the class, or the bulls of the faculty. When in addition to your own great powers, you are master of their little abilities as well, and society understands that though you cannot beat them in censure you can in practice—and that on their own principles—who will buy china of the peddlers when the crockery merchants have all that they offer, and on better terms?

But gentlemen, it is time that I had concluded my address. Accept my hearty congratulations for what I hear of your zeal and studiousness in the past, and for the hope which you have inspired in teachers and friends, touching your career in the future. May all their anticipations and yours be not only realized, but outdone in the event. With a steadfast purpose, a single and unchanging aim, the nobleness that deserves, the diligence that conquers success, may each of you reach the distant, difficult, but yet accessible goal, where the foremost of your models and guides have stood in other times; where so many of the living, and among them your own teachers, stand to-night, waiting the auspicious hour when they may lay upon your deserving brows the garlands that so worthily adorn their own.

